# Modelling the Relationship between the Nature of Work Factors and Driving Performance Mediating by Role of Fatigue

**DOI:** 10.3390/ijerph18136752

**Published:** 2021-06-23

**Authors:** Al-Baraa Abdulrahman Al-Mekhlafi, Ahmad Shahrul Nizam Isha, Nicholas Chileshe, Mohammed Abdulrab, Anwar Ameen Hezam Saeed, Ahmed Farouk Kineber

**Affiliations:** 1 Department of Management & Humanities, Universiti Teknologi PETRONAS, Seri Iskandar 32610, Perak, Malaysia; shahrul.nizam@utp.edu.my; 2 UniSA STEM, Scarce Resources and Circular Economy (ScaRCE), University of South Australia, Adelaide 5001, Australia; Nicholas.chileshe@unisa.edu.au; 3 Management Department, Community College of Qatar, Doha 00974, Qatar; abdulrabd@gmail.com; 4 Department of Chemical Engineering, Universiti Teknologi PETRONAS, Seri Iskandar 32610, Perak, Malaysia; anwar_17006829@utp.edu.my; 5 Department of Civil & Environmental Engineering, Universiti Teknologi PETRONAS, Seri Iskandar 32610, Perak, Malaysia; a.farouk.kineber@gmail.com

**Keywords:** nature of work, driving performance, work schedule, work activities, driving fatigue, oil and gas tanker driver, structural equation modelling

## Abstract

Driving fatigue is a serious issue for the transportation sector, decreasing the driver’s performance and increasing accident risk. This study aims to investigate how fatigue mediates the relationship between the nature of work factors and driving performance. The approach included a review of the previous studies to select the dimensional items for the data collection instrument. A pilot test to identify potential modification to the questionnaire was conducted, then structural equation modelling (SEM) was performed on a stratified sample of 307 drivers, to test the suggested hypotheses. Based on the results, five hypotheses have indirect relationships, four of which have a significant effect. Besides, the results show that driving fatigue partially mediates the relationship between the work schedule and driving performance and fully mediates in the relationship between work activities and driving performance. The nature of work and human factors is the most common reason related to road accidents. Therefore, the emphasis on driving performance and fatigue factors would thereby lead to preventing fatal crashes and life loss.

## 1. Introduction

Fatigue is a chronic biochemical issue that affects all drivers, and it is thought to be a major cause of traffic accidents. Fatigue has been linked to 10–20% of serious injuries, according to reports [1,2,3,4]. Sleep-related variables such as the circadian rhythm (i.e., time of day) and sleep restriction are strongly linked to driver fatigue [5]. Employers should be aware of their current fatigue rate, which would be linked to work design, prolonged periods of physical or mental effort, insufficient break time between shifts, and insufficient rest. The attention of drivers in driving duties can contribute immensely to the enhancement of their alertness performance [6]. Oil and gas truck drivers were one of the occupations with a significant risk of fatigue. As a result, safety and protection are critical, as the risk of an accident in this sector is high [7]; further, the severity of crashes of the heavy vehicle leads to huge casualties and losses of the property, as they often occur near or within residential areas [8,9]. Workplace safety is one of the most significant concerns that organizations must address since it is one of the most critical aspects in determining a company’s success. Accidents at work result in major business losses, such as injuries, asset damage, and productivity loss [10].

In the oil and gas transportation sector, work schedule and work activities are considered as risk factors for driving performance. Work in the schedule shifts has decreased drivers performance because of circadian rhythm disruptions [11]. According to Adams-Guppy [12], extended driving time is highly associated with the poor performance of drivers in the urban areas for professional drivers. Besides, there are many activities drivers have to perform besides driving, contributing to decreasing driving performance, such as filling fuel for the tanker, checking everything before moving, loading the order in the supplier terminal, downloading the order in customer stations, taking care of order invoices and cleaning the tanker when the driver comes back. Studies in the Malaysian oil and gas transportation sector have examined the effects of exhaustion-related psychological risk factors [13], psychological well-being and fatigue [14,15], perceived stress [16], and the assessment of fatigue by the psychomotor vigilance test [17]. Although these factors were important, however, the impact of the nature of work factors (work schedule and work activities) on driving performance and the mechanism of how fatigue mediates this relationship have been neglected. Currently, there is an urgent need to address the low performance among Malaysian oil and gas tanker drivers caused by the nature of work factors (work schedule and work activities) and driving fatigue.

This study aims to make up for the lack of empirical research on the effect of the nature of work factors on driving performance and to investigate the mechanism impact of driving fatigue on the relationship between the nature of work factors and driving performance in the Malaysian oil and gas transportation context. For this to happen, this study utilizes structural equation modelling by the partial least squares (PLS-SEM) approach. This study has focused on the nature of work as causal factors impairing the performance of oil and gas tanker drivers. Understanding the interaction between the performance of drivers and the factors influencing their performance will allow them to become part of the performance model development [18]. To ensure safe tanker journeys, the availability of this performance model will include a comprehensive measure of the driver’s performance [19]. Additionally, the performance assessment improves service quality, reduces risk degree, and prevents the occurrence of accidents.

## 2. Literature Review and Hypotheses Development

In general, performance can be well-defined as the capacity of an individual to perform a specific task over some time [20]. Therefore, driving performance can be defined as the effectiveness of a driver to complete the driving duty. The effectiveness can be concerning vigilance, reaction time, and the attention of drivers, and can be measured when the drivers perform their duties [20,21,22]. The need for improvement can be rationally assessed and determined by measuring human performance. In the early stages of the design process, the performance development factors that are in line with the workers’ characteristics, needs and knowledge should be considered [23]. Delayed improvements may occur if the organization’s management does not understand human performance [24].

The performance levels of an employee often are directly related to the characteristics of the employee and the nature of their work. The essence of the work of an employee is better described as the sort of work they carry out. This could include the everyday simple activities performed as part of a job and other non-routine activities [25]. Thus, the nature of driving work includes driving, shifting activities, monotone driving, and environmental interaction [26,27]. In the current study, the essence of a driver’s job is typical of these tasks (attention, vigilance, reaction time) [20,21,22], driving fatigue influences the reaction abilities of drivers [28,29], and can be summed up by the two guiding forces impacting the performance, work schedule, and work activities.

### 2.1. Work Schedule

Employees working in shift systems often have physical and psychological health issues related to working in shifts [30,31]. According to previous research, forming the risk index for work schedule, clear patterns were identified: relative occupational risk (i) increased by 18% for a shift in the afternoon and evening and 30% for night shift, compared to a shift in the morning; (ii) it was 6% higher in the second night, 17% higher in the third and 36% higher in the fourth night, compared to the first night shift, and a similar though slower pattern was observed for day shifts of 2%, 7%, and 17%, respectively, increased the risk of accidents; (iii) the rise was 13% for 10 h shifts and 27% for 12 h shifts compared to 8 h shift; and (iv) recorded increases essentially linearly with time spent on the driving such that after 90–119 min, the possibility of accidents is high, relative to the first 30 min [32]. Heavy vehicles are known to be a very dangerous occupation due to long periods of driving and poor physical activities when driving. The prevalence of fatigue increases due to these risk factors associated with shift work and irregular sleep patterns. Subsequently, fatigue is associated closely with stress and resulting in low physiological and psychological [33]. Research was conducted in 2005 on the connection between workability and working time. The study showed that decreased workability between day workers is higher than between night workers, as the Work Ability Index indicated (WAI) [34], and 63% of construction workers were suffering from poor quality of sleep and daytime dysfunction and physical fatigue [35,36]. Shifting, including work at night, is regarded as a major risk factor. A study found that employees are at enhanced risk of accidents during successive night shifts [37]. Besides, commuting home by car after a night shift is another safety hazard for employees in the shift [38]. The literature also indicates that those employees who work a night shift have increased fatigue and drowsiness [39], and both are considered to be important risk factors for road accidents [5,40,41]. The literature also suggests that working a night shift is linked with increased driver’s sleepiness and self-reported driving impairments [42].

Regarding the industry, a classic study by Bjerner [43] noticed that shift time and length of the shift are other variables that can affect the safety of drivers. According to Rogers [44], a shift of ten hours and twelve hours are tighter, and the drivers are thus more likely to fall asleep after a night shift. Reduced sleepiness and poor vigilance of drivers are connected more specifically with longer journey time (>35 min) and decreased sleep time (6 h). Gillberg [45] demonstrated that driving at night was slow with greater speed variability, and subjective alertness was much smaller.

A shift work disorder or non-standard shift is a fundamental sleep disorder in the Circadian Rhythm Sleep Disorders group [46]. It is described as extreme sleep and/or insomnia that is temporally correlated with a work schedule. Drake and colleagues proposed that the prevalence of a shift work disorder is observed in at least 10% of those working in the night and rotating shifts [47]. A shift work disorder in primary care has been incorrectly diagnosed, most possibly due to the lack of standardized screening equipment [48,49]. It is also not well-known that a driver may be susceptible to adverse health and safety consequences of work, partly because a shift work disorder lacks an effective monitoring tool. Therefore, in this study, three types of work schedules were applied to assess the performance of oil and gas drivers: day shift, night shift, and non-standard shift. Therefore, the previous literature review hypothesized that:

**Hypothesis** **1.** *Work schedule has a significant impact on driving performance.*

**Hypothesis** **2.** *Work schedule has a significant impact on driving fatigue.*

### 2.2. Work Activities

The activities of humans are one of the significant factors that affect human performance [50]. Work activities are referred to as the task or job performed by an employee or driver or an individual’s daily work activities demanded by a superior [51].

Job demand refers to the psychological, physical, social or organizational elements of the work, which require continuous physical or psychological efforts or abilities and therefore involve certain physiological and/or psychological costs [52,53,54]. Job demand can become stressful at work if the demand and abilities are not balanced [52]. People suffer from work-related stress when their employment requirements exceed their mental and physical capabilities, and hence are dangerous or harmful to their well-being. There was also an important social impact of workers’ exhaustion, such as health care costs and lack of productivity [55,56].

The consequences of driving tasks have been addressed in several studies [57,58,59]. The driver should have dynamic control and takes real-time decisions; also, drivers must be aware of the signal, regulations and safety messages during a journey [60]. A monotonous, long-period driving task can lead to a driver’s fatigue and reduce his/her alertness and ability to monitor incidents and information collected during the trip, hence reacting to them [7,61]. Thus, in the present study, work activities, including job demands and driving tasks, were determined to be the important factors affecting the driving performance of oil and gas tanker drivers. Therefore, the previous literature review hypothesized that:

**Hypothesis** **3.** *Work activities have a significant impact on driving performance.*

**Hypothesis** **4.** *Work activities have a significant impact on driving fatigue.*

### 2.3. Fatigue as a Mediation

A mediation model aims to define and clarify the process that underlies an observed relationship between independent and dependent variables through the incorporation of a hypothesis third variable known as a mediator variable (intermediary variable, mediating variable or intermediating vary) [62].

Many studies have found that fatigue is a danger to drivers, which decreases their performance and causes road crashes [7,8,63]. Heavy-duty drivers are more likely to cause human and economic losses in a road accident [63,64]. To devise and introduce safe and efficient interventions to reduce mortality, it is relevant to know the accidental history of distinct organizations with distinct drivers’ employment types [65]. Fatigue results in the deterioration of mental and physical health, and in the case of drivers, fatigue is the cause of 20% of the total road crashes [66]. Besides, Meyer [67] have examined the link between the indirect and direct present exposure of stress, and multiple results (i.e., satisfaction with empathy, and employment satisfaction) were examined by a non-parametrical bootstrapping technique, both for indirect and direct pre-existing exposure of stress [68,69].

In Malaysia, the proportion of accidents are significantly greater than 20%, as fatigue impedes human effectiveness to work efficiently [70]. The Malaysian Institute of Road Safety Research reported about 34.7% of accidents of tanker drivers because of fatigue due to poor quality of sleep [71]. Driving fatigue was chosen as a mediator to examine the relationship between the nature of work and driving performance among oil and gas tanker drivers in Malaysia. Therefore, in this study, driving fatigue is selected theoretically as a mediator variable for studying indirect relationships. To verify the mechanism of the mediating role of driving fatigue on the relationship between nature of work and driving performance, therefore, this study proposes the following hypotheses:

**Hypothesis** **5.** *Driving fatigue has a significant impact on driving performance.*

**Hypothesis** **6.** *Driving fatigue mediates the relationship between work schedule and driving performance.*

**Hypothesis** **7.** *Driving fatigue mediates the relationship between work activities and driving performance.*

**Figure 1:**
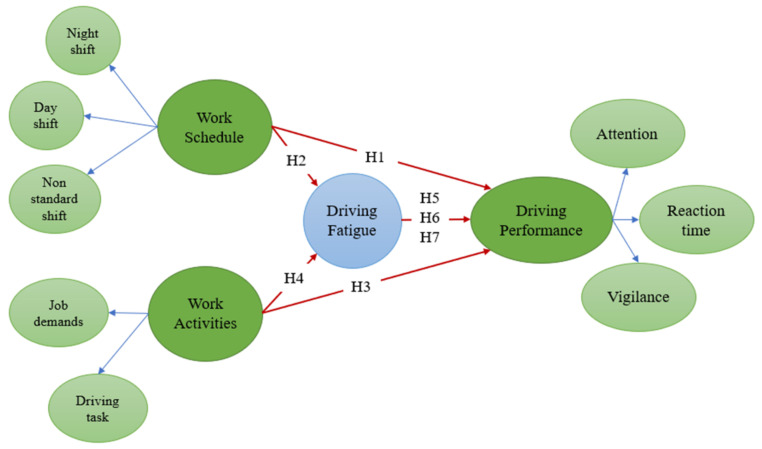
Driving performance hypothetical model.

In the theoretical underpinning, the current study depends on the effort–compensation theory to create the conceptual framework [72]. This theory suggested that, in long-haul driving, stress can result from many factors, such as job demands, driving tasks, and shift schedule. This stress will directly impact the alertness of drivers, leading to lower performance levels. Thus, maintaining attention requires constant self-regulation by the driver. The driver must choose between the personal costs (i.e., effort or exertion) and the benefits (i.e., extrinsic and intrinsic rewards) to maintain vigilance over time [73]. Hence, based on this theory, we proposed the following framework (see Figure 1).

## 3. Research Approaches

### 3.1. SEM (Structural Equation Modeling)

SEM is known as a multivariate method for analyzing the accuracy of competing hypotheses and collected samples concerning a concept and theory [74,75]. Covariance-based structural equation modelling (CB-SEM), and partial least squares structural equation modelling (PLS-SEM) are the two major methods for SEM [76,77]. In the context of defining the relationship between items and constructs for the researchers, PLS-SEM is more flexible than CB-SEM [78]. PLS-SEM performs very well in any given sample size but it should meet the minimum criteria of the sample size which makes it possible for variables to be developed with complicated impacts on particular aspects of the model. PLS-SEM deals with constructs or latent variables (composites) which can operate with the measuring models in mode A (reflective), and mode B (formative). Thus, the SEM technique is commonly utilized by researchers [79]. The SEM approach has the following advantages: first, SEM can be utilized to reliably estimate complex hypothesis models based on several observations [80]. Secondly, SEM works well, especially for highly complex models composed of many numbers of latent variables and indicators. It aims, therefore, to obtain models as parsimonious as possible [80]. SEM has currently been used in many areas of social science analysis effectively; for instance, hospitality management [81], construction industry [82,83,84,85], competitive performance [86], the environment and organization [87].

As a result, the PLS-SEM method was utilized to evaluate the seven proposed hypotheses in this study. For this to happen, the Smartpls v3.2.1 (SmartPLS GmbH, Bönning-stedt, Germany) program was used to test the measurement model’s fitting index and path analysis for the model [88]. To test common method bias, SPSS version 25.0 software was utilized to test Harman’s single factor. In order to test multicollinearity, the variance inflation factor (VIF) was utilized to assess multicollinearity issues [88].

### 3.2. Sampling and Data Collection

The current study utilized a Likert scale which has been used in several studies [89,90], with 43 items as the survey questionnaire [46,91,92,93,94,95,96]. The structure of the study variables is shown in Table 1. In Supplementary Table S1, items of the study questionnaire are shown.

This study applied a stratified random sampling technique to choose the sample from the population of the study. Stratified random sampling is defined as a process of stratification or segregation which is usually followed by randomly selecting the subjects from each stratum [97]. A total of 357 questionnaires were distributed to oil and gas tanker drivers from most of the regions in Malaysia. Distributing and collecting the questionnaire was carried out manually, and the survey time was from 2019–2020. After invalid surveys were excluded, 307 valid surveys were collected; the response rate was 85.9%. The evaluated model consists of five latent variables and 43 observable variables. According to Clemente et al. [98] and Maccallum and Bryant [99], this sample size meets the standard on the sample size in SEM; the minimum sample size was assessed using Gpower software v3.1 University of Dusseldorf, Dusseldorf, Germany. The sample size was estimated with α of 0.05 and power of 1-β = 0.80, and the outcomes specified a sample of (279) as a minimum, that is smaller than the current study included (*n* = 307). Table 2 shows the descriptive statistics for the sample.

Table 2 shows the proportion of males and females 306 (99.7%) and 1 (0.3%); the survey gender structure showed a higher proportion of men than women, which is in line with the characteristics of the Malaysian oil and gas transportation sector, as the drivers of heavy vehicles are not typically females. From the frequencies of age groups of the sample, 14.7% of them are in the age group 20–29 years old, and 48.2% of total respondents are between 30–39 years, while 26.4% of them are in the age group of 40–49 years and 10.1% of total respondents are in the age group of 50–59 years, while 0.7% were in a group of 60 years and above. Thus, age composition samples were mostly middle-aged and young, which is in line with the requirement to work as a driver in the oil and gas transportation industry. In terms of the marital status of respondents, 84.4% were married, 12.7% were single, and 2.9% were separated. In terms of the background of education, the majority of the respondents 83.7% had secondary education. Meanwhile, 12.7% of the respondents had college diploma degree qualifications. Furthermore, respondents who had a primary education represent 2.6%, and 1% graduate/postgraduate education. Therefore, the proportion of secondary education was very high, which is in line with the criteria of employment in the oil and gas transportation sector.

## 4. Results

### 4.1. Tests of Model Fit

To confirm the validity of the PLS-SEM model, the *GoF* value was measured according to the guidelines laid down by Wetzels [100] who argued that the closer the *GoF* to 1, the better the fit of the model under consideration *GoF* = (0 < *GoF* > 1) will be. The *GoF* value for the model was precisely determined with the following formula:

Equation (1) *GoF* value for the model

(1) $$GoF=\sqrt{(\bar{R^{2}}x\bar{AVE)}}$$

The observed *GoF* value was 0.685 in this study (average *R*^2^ is 0.831, mean *AVE* 0.825). *R*^2^ is the average *R*^2^ value for the endogenous constructs, *AVE* is the average variance extracted. The baseline values of *GoF* (small = 0.1, medium = 0.25, high = 0.36) were compared. This showed a sufficient global validity of the PLS model.

#### 4.1.1. Analysis of Reliability

Reliability and validity tests were used to assess the measured model. Four indicators, i.e., standardized indicator loadings (SIL), Cronbach’s alpha (CA), composite reliability (CR), and average variance extracted (AVE), were tested during the reliability test. (i) The values of CA [101], CR [102], SIL [103] must be higher than 0.70. (ii) AVE value, the AVE should be above 0.5 for every construct [104,105]. The degree of explained variance of the endogenous variables is represented by the determination coefficient (R**^2^**) [78]. The explanatory power of a structural model can be determined by using the R**^2^** [106]. The R**^2^** must be acceptable, with weak, medium, and substantial values of 0.25, 0.50, and 0.75, respectively, for target constructs [107]. The test results for reliability and R**^2^** are shown in Table 3.

Table 3 reveals that the value of SIL was from 0.700 to 0.989 (above 0.700), the value of CA was from 0.897 to 0.772 (over 0.700), the value of CR ranged from 0.898 to 0.984 (above 0.700), the value of AVE was from 0.680 up to 0.864 (above 0.500) and the value of R***^2^*** was from 0.748 to 0.831. (Above 0.50).

#### 4.1.2. Discriminant Validity

To complete the discriminant validity test, the heterotrait–monotrait ratio (HTMT) of correlations was applied [108], as seen in Table 4. The heterotrait–monotrait ratio test of correlations is superior in comparison with Fornell–Larcker criterion and (partial) cross-loadings. The recommended threshold (0.9) was used as the criterion.

Therefore, the results of the measured model demonstrated the high reliability, convergence validity, discrimination validity and verified the constructures were statistically diverse.

### 4.2. Structural Model Assessment

#### 4.2.1. Direct Effect

**Figure 2:**
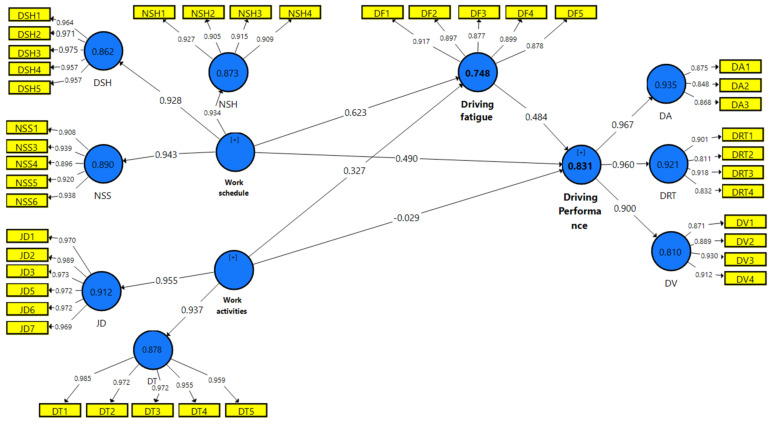
Measurement model test.

For hypotheses testing, seven hypotheses were tested, five of which were tested for direct relationships, and the rest were tested mediating relationships. In a direct relationship, two constructs were under nature of work (work schedule, work activities) as independent variables, driving performance in the structural model framework was one dependent variable, and driving fatigue was the mediating variable. For these higher-order constructs (work schedule, work activities, and driving performance), to estimate the latent variables, a repeated indicator approach was used. Effect size was used to measure the significant impact of an independent variable on a dependent variable [109]. The effect size (F2) of 0.02, 0.15, and 0.35 are regarded as small, moderate, and strong [110]. Table 5 indicates that the effect sizes were moderate and strong between variables. Following the application of the consistent PLS algorithm, Figure 2 shows the outcomes of measured model tests.

First, we maintained both missing values and the PLS-SEM algorithm settings, as with the original model estimate. The next choice to run the 5000 bootstrap samples was chosen for “Sign No Changes” and “Complete Bootstrapping.” Finally, in the bootstrapping procedure, the bias acceleration and correction process was used, and the advanced settings conducted a one-tailed 0.05 significance level test. Table 5 indicates that four hypotheses (i.e., H1, H2, H4, H5) are supported, while one is not supported (H3). There is a strong impact size for three hypotheses supported (i.e., H1, H2, and H5), with a moderate effect on the supported hypothesis (H4).

#### 4.2.2. Mediation Effect Analysis

The present study used the bootstrapping approach to confirm the mediating effect [69]. This approach has been used and suggested by several researchers for studies that aim to evaluate such indirect effects [68]. Besides, bootstrap results are stated to have probability estimates that are more accurate, because this method assists in overcoming mediation problems and lacks normal distribution of the mediator and outcome variables [111]. Thus, the application of this approach is supported by two reasons. The first reason is that it offers a good means to test the significance and confidence intervals in various situations. Another reason is that the use of this method does not need many assumptions. Therefore, the results obtained through this method are more accurate [62]. Table 6 shows the findings of indirect effect, all mediating hypotheses were supported. See Supplementary Table S2 for the full table of mediating impact analysis.

## 5. Discussion

The driving performance was derived from the literature written in different fields. In this study, seven hypotheses were tested, five of which were critical predictors for direct relationships, while the rest used driving fatigue as a mediating relationship on driving performance. However, Table 5 indicates that the structure of work activities construct failed to predict the driving performance, with four direct hypotheses of statistical significance (H1, H2, H4, and H5). Otherwise, the findings of this analysis revealed a direct hypothesis (H3) that is statistically not significant.

The work schedule has been evaluated from three viewpoints: day shift, night shift, and non-standard shift. The work schedule explored the drivers’ understanding of the effect on their performance during the driving processes. As the drivers spend most of their time on the road, one might argue that, either because of their shift time or non-standard shift, there’s a probability that the drivers will be prone to fatigue or the impairment of physical or mental functions during their driving duty shift. This increases the possibility of road accidents. This study found that in (H1), work schedule affects the driving performance (β = 0.490, *t* = 8.782, *p* < 0.000); likewise, in hypothesis (H2), this study found that work schedule affects driving fatigue as well (β = 0.623, *t* = 13.244, *p* < 0.000). This finding is aligned with prior findings reported in several prior studies [34,37,48,91]. All these studies reported that the work schedule has a significant impact on driving performance and driving fatigue.

Work activity is represented as one of the core elements in the day system that affects the driver’s performance in the oil and gas transportation sector. Since the drivers’ work specializes in the transportation of oil and gas, the drivers need to carry out many activities besides their usual driving duty, as mentioned in the introduction. However, the outcomes did not find a significant influence between work activities and driving performance in the context of Malaysian oil and gas tanker drivers (β = −0.029, *t* = 0.716, *p* > 0.05 = 0.474). Surprisingly, the study revealed that the work activities (H3) (job demand and driving task) had an insignificant impact on driving performance. Further analysis has found that tanker drivers rely rather more on immediate demand during driving duties than management demand. Demands from the superiors and management were expected to be the normal daily routine for the drivers; however, because most of the demands were part of their normal routine, the tanker drivers were required to only follow their everyday duty activities. The drivers’ daily activities are also not stressful to undertake when the activities are balanced and there are no high demands [52].

In contrast, in (H4), work activities had a significant impact on the fatigue of the tanker drivers (β = 0.327, *t* = 6.532, *p* < 0.000). This indicates that when tanker drivers are driving, they must be careful and mindful of the surroundings on the road [112]. Kecklund [113] has provided similar reasoning on the relationship of driving activities with vigilance. The driver must be alert to the surrounding environments including signals, illegal trespassers, vehicle distance, and should also ensure that he/she is focused and is paying attention, since driving the tanker for a long journey is monotonous and boring task [113].

Fatigue in (H5) also had a significant influence on the tanker drivers’ driving performance (β = 0.484, *t* = 7.549, *p* < 0.000). This result is not surprising because fatigue has a significant correlation with the drivers’ alertness, attention, and reaction time [26,27,114]. Other researchers have also linked fatigue to have a major effect on the driver’s performance [115,116]. This also decreases the level of alertness during driving, thus making it difficult to concentrate on signs, signals, and the surrounding environment. Over time, if fatigue is left unabated, the driver will fall asleep [112,117,118]. Similarly, previous experiments have found that long driving is monotonous and boring, thus inducing fatigue and causing the driver to fall asleep [26,27].

A mediating effect is developed when there is an intervention of a third variable or construct with two other related constructs [78]. The present study used a bootstrapping method to confirm the mediating effect. The driving fatigue constructs mediate two relationships between the nature of work factors (work schedule, work activities) and driving performance constructs [69]. Based on the results in Table 4, it can be seen that there is a significant mediating impact of driving fatigue between IV and DV. The findings of the analysis thus confirmed the two hypotheses (H6, H7) to be statistically significant.

In (H6) indirect effect, Preacher and Hayes [69] indicated that 0.302, 95 percent boot CI: (LL = 0.192, UL = 0.411) does not straddle a 0 in between, which indicates there is a mediation. It can be concluded from the statistical finding that there is a significant mediation effect of the DF between variables. Besides, there is a statistically significant direct effect between WS (IV) and DP (DV) (β = 0.490, *t* = 8.782, *p* < 0.000), as shown in Table 5. Accordingly, DF mediates the relationship between WS and DP, and therefore, H6 is supported. The result of the study showed that driving fatigue mediated the relationships between work schedule and driving performance among Malaysian oil and gas tanker drivers. This implies that the work schedule negatively affects the performance of the driver, such as vigilance and attention [30,31]. As a result, this contributes to performance degradation through driver fatigue [26]. In other words, this explanation considers the effects of the shift time on the performance of drivers as mere reactions of the negative drivers’ behaviors toward arranging their shift time and rest time. Therefore, these results are in agreement with the outcomes of previous relevant studies [119,120].

Moreover, in (H7), Preacher and Hayes [69] imply that the 0.158, 95 percent boot CI: (LL = 0.080, UL = 0.237) does not straddle a 0 in between, which implies that there is an existence of mediation. Therefore, this study can conclude that there is a significant mediation effect of the DF between variables. However, the direct effect between WA (IV) and DP (DV) is not significant (β = −0.029, *T*-value = 0.716, *p*-value = 0.474). Accordingly, DF mediates the relationship between WA and DP, thus indicating that H7 is supported. The result of the study showed that driving fatigue mediates the relationships between work activities and driving performance among Malaysian oil and gas tanker drivers. This implies that the work activities significantly affect the performance of the driver, such as vigilance and attention [121]. This further contributes to performance degradation through driver fatigue [122]. In other words, this explanation considers the effects of daily work activities on the performance of drivers through driver fatigue and concludes that there is no direct effect between the activities and performance.

## 6. Conclusions

According to the results and discussion above, this paper investigated the mediating role of driving fatigue in the relationship between the nature of work factors (work schedule and work activities) and driving performance. The results indicate that the relationship between work activities and driving performance has not been significant. The work schedule, however, was found to affect driving performance and driving fatigue significantly. Additionally, fatigue has been shown to affect driving performance significantly. Moreover, the study demonstrated the mediating role of driving fatigue as a mechanism that explains the relationships between work schedule, work activities, and driving performance. The outcomes of this study may also have theoretical and practical implications. From a theoretical point of view, the present study extends the effort composition theory by developing a more comprehensive mediating model that incorporates various driving fatigue perspectives. Furthermore, this study contributes to the existing body of knowledge by investigating the mediating role of fatigue in the relationship between the nature of work factors and driving performance. Otherwise, in practical implications, this study is among the first studies to provide empirical evidence to oil and gas transportation policymakers to support their decision making regarding the design of drivers’ work schedules and determining the activities of drivers to avoid driving fatigue. Moreover, this study provides information on the importance of each factor considered in this study, that encourages the direct supervisors of drivers to set assessment criteria and taking into consideration the importance of the nature of work factors and the degree to which each factor leads to a decrease or increase in driving performance.

Despite the contributions of this study, the inherent limitation related to the data collection method is acknowledged. As this study depended on oil and gas tanker drivers to collect data to examine their perspective toward study variables, this does not reflect a deep understanding of the study issue from all parties involved; therefore, it is recommended for a qualitative method to be employed to identify other elements relating to driving fatigue and driving performance from supervisors’ and administrator’s perspectives in the oil and gas transportation companies to obtain precious results. Considering the outcomes of this study, other interesting issues that can be studied in the future investigation are safety culture and psychosocial hazards among oil and gas tanker drivers.

## Figures and Tables

**Table 1:** Structure of the study instrument.

Constructs	Dimensions	No. of Items	Reference
Work schedule		15	[46,91]
	Night shift (NSH)	4	
	Day shift (DSH)	5	
	Non-standard shift (NSS)	6	
Work activities		12	[92,93]
	Job demand (JD)	7	
	Driving Task (DT)	5	
Driving performance		11	[94,95]
	Attention (DA)	3	
	Reaction time (DRT)	4	
	Vigilance (DV)	4	
Driving fatigue (DF)		5	[96]

**Table 2:** Demographic variables (*n* = 307).

Construct	Category	Frequency	Percentage
Gender	Male	306	99.7%
	Female	1	0.3%
Age	20–29 years	45	14.7%
	30–39 years	148	48.2%
	40–49 years	81	26.4%
	50–59 years	31	10.1%
	60 years and above	2	0.7%
Marital	Single	39	12.7%
	Married	259	84.4%
	Separated	9	2.9%
Education	Graduate/Postgraduate	3	1%
	College/Polytechnic	39	12.7%
	Secondary	257	83.7%
	Primary	8	2.6%

**Table 3:** Validity, reliability, and value of R***^2^***.

Constructs	Path Relationships	SIL	CA	CR	AVE	R*^2^*	
						Value	LEP
WS			0.976	0.898	0.746	-	-
NSH1 ←WS		0.927					
NSH2 ← WS		0.905					
NSH3 ← WS		0.915					
NSH4 ← WS		0.909					
DSH1 ← WS		0.964					
DSH2 ← WS		0.971					
DSH3 ← WS		0.975					
DSH4 ← WS		0.957					
DSH5 ← WS		0.957					
NSS1 ← WS		0.908					
NSS3 ← WS		0.939					
NSS4 ← WS		0.896					
NSS5 ← WS		0.920					
NSS6 ← WS		0.938					
NSS1 ← WS		0.908					
WA			0.982	0.984	0.846	-	-
JD1 ← WA		0.970					
JD2 ← WA		0.989					
JD3 ← WA		0.973					
JD5 ← WA		0.972					
JD6 ← WA		0.972					
JD7 ← WA		0.969					
DT1 ← WA		0.985					
DT2 ← WA		0.972					
DT3 ← WA		0.972					
DT4 ← WA		0.955					
DT5 ← WA		0.959					
DF			0.937	0.952	0.799	0.748	Medium
DF1 ←DF		0.917					
DF2 ← DF		0.897					
DF3 ← DF		0.877					
DF4 ← DF		0.899					
DF5 ← DF		0.878					
DP			0.953	0.959	0.680	0.831	Substantial
DA1 ← DP		0.875					
DA2 ← DP		0.848					
DA3 ← DP		0.868					
DRT1 ← DP		0.901					
DRT2 ← DP		0.811					
DRT3 ← DP		0.918					
DRT4 ← DP		0.832					
DV1 ← DP		0.871					
DV2 ← DP		0.889					
DV3 ← DP		0.930					
DV4 ← DP		0.912					

SIL: standardized indicator loadings, CA: Cronbach’s alpha, CR: composite reliability, AVE: average variance extracted, LEP: level of explanatory power, WS: work schedule, NSH: night shift, DSH: day shift, NNS: non-standard shift, WA: work activities, JD: job demand, DT: driving task, DF: driving fatigue, DP: driving performance, DA: driving attention, DRT: driver reaction time, DV: driver vigilance.

**Table 4:** Values of heterotrait–monotrait ratio (HTMT).

	DA	DRT	DSH	DT	DV	DF	JD	NSH	NSS
DA									
DRT	0.408								
DSH	0.833	0.614							
DT	0.614	0.578	0.559						
DV	0.779	0.723	0.754	0.652					
DF	0.717	0.674	0.787	0.748	0.603				
JD	0.600	0.558	0.519	0.801	0.563	0.664			
NSH	0.643	0.472	0.826	0.645	0.821	0.767	0.622		
NSS	0.758	0.736	0.815	0.561	0.813	0.804	0.523	0.311	

**Table 5:** Direct effect summary.

Hypotheses	H1	H2	H3	H4	H5
Path Relationships	WS → DP	WS → DF	WA → DP	WA → DF	DF → DP
Path coefficient (β)	0.490	0.623	−0.029	0.327	0.484
Standard Error	0.056	0.047	0.040	0.050	0.064
F2 Value	0.449	0.948	0.002	0.261	0.350
Effect	Strong	Strong	No Effect Size	Moderate	Strong
*t* Values	8.782	13.244	0.716	6.532	7.549
*p* Values	0.000	0.000	0.474	0.000	0.000
Significance level *** *p* < 0.001	***	***	-	***	***
Result	Supported	Supported	Not Supported	Supported	Supported

**Table 6:** Analysis of mediation.

Relationship		Indirect Effect			Bootstrapped Confidence Interval		Decision
		Path Coeff	SE	*t*-Value	95% LL	95% UL	
H6	WS-DF-DP	0.302 **	0.056	5.385	0.192	0.411	Partial mediation
H7	WA-DF-DP	0.158 **	0.040	3.957	0.080	0.237	Full mediation

Note: ** = *p* < 0.01, LL: lower level, UL: upper level.

## Data Availability

The data are available for those who want to see it with justified reasons. Kindly contact the corresponding author.

## Supplementary Materials

The following are available online at https://www.mdpi.com/article/10.3390/ijerph18136752/s1, Table S1: Questionnaire items, Table S2: Analysis of mediation.
