# Supplementary material for: Modelling the Relationship between the Nature of Work Factors and Driving Performance Mediating by Role of Fatigue
Source: Int J Environ Res Public Health. 2021 Jun 23;18(13):6752. doi: 10.3390/ijerph18136752 (PMC8268994; doi:10.3390/ijerph18136752)
Supplement: Supplementary file 1 [file ijerph-18-06752-s001.zip › Supplementary Information1.pdf]

International Journal of
Environmental Research and Public Health

MDPI

Supplementary Information

Table S1. Questionnaire items

Construct	CODE	Item	References
**Work schedule**			
Night shift	NSH1	I have no experience sleepiness while driving.	[46, 91]
	NSH2	I have no experience decreased in my physical or mental functioning during the time I were awake.	
	NSH3	I have no experience sleepiness while commuting to my home after working the night shift.	
	NSH4	During night shift, I do not experience doze off while driving.	
Day shift	DSH1	I don't have sleep problems such as snoring or obstructive sleep apnea	
	DSH2	During the day shift, I do not experience doze off while driving.	
	DSH3	During the day shift, I have no experience decreased in my physical or mental functioning during the time I were awake.	
	DSH4	During the day shift, I have no experience sleepiness while driving.	
	DSH5	I have no experience the time delay in getting to sleep at bedtime	
Non-standard shifts	NNS1	The overall amount of sleep was somewhat insufficient.	
	NNS2	I have no experience sleepiness while driving.	
	NNS3	I do not have a problem falling asleep at bedtime	
	NNS4	I do not have a problem with waking up too early and not being able to get back to sleep	
	NNS5	During my break, I have no experience sleepiness during the time I were awake.	
	NNS6	During my break, I do not have a problem falling asleep at bedtime.	
**Work activities**			
Job demand	JD1	I complete the same basic activities most of the time.	[92, 93]
	JD2	I deal with problems which are difficult to solve.	
	JD3	I am rarely often having spare time in my work.	
	JD4	I am challenged by my job.	
	JD5	An error on my part could cause a safety incident.	
	JD6	The more mental concentration required than you would like.	
	JD7	I have to drive for long periods (2 hours and more).	
Driving task	DT1	Driving is not running according to schedule	
	DT2	Becoming very drowsy while driving	
	DT3	Feel boredom and monotony of the job	
	DT4	Do you drive during rush hours?	
	DT5	Being issued time-table alterations on the day of travelling	
**Driving performance**			
Attention	DA1	Operating entertainment systems do not distract me from driving (e.g., playing radio).	[94, 95]
	DA2	Operating navigation systems do not distract me from driving.	
	DA3	I sometimes push the wrong pedal	
Reaction time	DRT1	My reactions are faster than they used to be (e.g., braking in an emergency).	
	DRT2	I sometimes cannot judge my speed.	
	DRT3	I have no difficulty judging the speed of oncoming vehicles.	

	DRT4	I have no trouble judging the distance from the vehicle in front.	
Vigilance	DV1	I have no difficulty with identifying and reading road signs.	
	DV2	I sometimes cannot hear the horns of other vehicles/sirens from emergency vehicles	
	DV3	Sometimes my speedometer is hard to read during the daytime.	
	DV4	Sometimes my speedometer is hard to read during the night time	
Driving fatigue			
Driving fa-tigue	DF1	Do you have to drive when you are tired?	[96]
	DF2	Do you drive when you have a minor illness like a cold?	
	DF3	Drive after not having much sleep?	
	DF4	Drive for longer than 2 hours without a break?	
	DF5	Drive after working for extended periods of time?	

Table S2. Analysis of mediation

	Relationship	Path(A)		Path (B)		Direct effect (c)		Indirect effect			Bootstrapped Confidence Interval		Decision
		Path Coeff	t-value	Path Coeff	t-value	Path Coeff	t-value	Path Coeff	SE	t-value	95% LL	95% UL	
**H6**	WS-DF-DP	0.623**	13.24	0.484**	7.549	0.490**	8.782	0.302**	0.056	5.385	0.192	0.411	Partial mediation
**H7**	WA-DF-DP	0.327**	6.532	0.484**	7.549	-0.029	0.716	0.158**	0.040	3.957	0.080	0.237	Full mediation
